# Restless legs syndrome in Parkinson disease: Clinical characteristics, abnormal iron metabolism and altered neurotransmitters

**DOI:** 10.1038/s41598-017-10593-7

**Published:** 2017-09-05

**Authors:** Ying-Shan Piao, Teng-Hong Lian, Yang Hu, Li-Jun Zuo, Peng Guo, Shu-Yang Yu, Li Liu, Zhao Jin, Hui Zhao, Li-Xia Li, Qiu-Jin yu, Rui-Dan Wang, Sheng-Di Chen, Piu Chan, Xiao-Min Wang, Wei Zhang

**Affiliations:** 1 Department of Geriatrics, Beijing Tiantan Hospital, Capital Medical University, Beijing, 100050 China; 2 Department of Neurology, Beijing Tiantan Hospital, Capital Medical University, Beijing, 100050 China; 3 China National Clinical Research Center for Neurological Diseases, Beijing, 100050 China; 4 Key Laboratory for Neurodegenerative Disorders of the Ministry of Education, Capital Medical University, Beijing, 100069 China; 5 Center of Parkinson Disease, Beijing Institute for Brain Disorders, Beijing, 100069 China; 6 Beijing Key Laboratory on Parkinson Disease, Beijing, 100053 China; 7 Department of Neurology, Ruijin Hospital Affiliated to Shanghai Jiaotong University School of Medicine, Shanghai, 200025 China; 8 Department of Neurobiology, Beijing Xuanwu Hospital, Capital Medical University, Beijing, 100053 China; 9 Department of Physiology, Capital Medical University, Beijing, 100069 China

## Abstract

Relationships among clinical characteristics, iron metabolism and neurotransmitters in Parkinson disease (PD) patients with restless legs syndrome (RLS) remains unclear. We divided 218 patients into PD with and with no RLS (PD-RLS and PD-NRLS) groups by RLS-rating scale (RLS-RS) score. Motor and non-motor symptoms were rated by related scales. Iron and related proteins, and neurotransmitters in cerebrospinal fluid (CSF) and serum were measured. PD-RLS frequency was 40.37%. PD-RLS group had longer duration, higher stage and scores of motor symptoms, depression, anxiety, sleep disorders, fatigue and apathy, and increased transferrin and decreased iron, ferritin, dopamine (DA) and 5-hydroxytryptamine (5-HT) in CSF. In CSF of PD-RLS group, RLS-RS score was positively correlated with transferrin level and negatively correlated with iron and ferritin levels; RLS-RS score was negatively correlated with DA and 5-HT levels; transferrin level was negatively correlated with DA and 5-HT levels, and ferritin level was positively correlated with DA level. In serum, PD-RLS group had decreased iron and transferrin levels, which were negatively correlated with RLS-RS score. PD-RLS was common and severer in motor and some non-motor symptoms. Iron deficiency induced by its metabolism dysfunctions in peripheral and central systems might cause PD-RLS through decreasing brain DA and 5-HT.

## Introduction

Parkinson disease (PD) is a common neurodegenerative disease in the elderly population with motor symptoms, including bradykinesia, resting tremor, rigidity and postural and gait instability. Recent advances in pathology suggest a variety of non-motor symptoms in PD patients, in which, restless legs syndrome (RLS) is the common one. RLS is characterized by a discomfort in both legs and strong desire to move them. It starts when patients are at rest, alleviates when patients are moving and exacerbates at night specifically^1^. RLS is found to be an independent factor affecting sleep quality, and therefore seriously compromises quality of life for PD patients^2^. However, it is not well known about the clinical characteristics of RLS, the relationship between RLS and motor symptoms or other non-motor symptoms of PD yet.

Both PD and RLS are related to abnormal iron metabolism by the increasing investigations. In PD patients, results from a variety of histopathological studies and autopsy quantitative tests revealed an excessive iron depositions in substantia nigra (SN)^3, 4^, which was furtherly confirmed by transcranial sonography^5^. Over iron deposition in SN was associated with abnormal iron metabolism in brain, excessive intake of exogenous iron, gene mutations of iron metabolism and damage of blood-brain barrier. Excessively accumulated iron led to the degeneration and death of dopaminergic neurons in SN and promoted development and progression of PD through multiple mechanisms involving oxidative stress, neuroinflammation, etc^6^. In RLS patients, the levels of iron and ferritin in cerebrospinal fluid (CSF) were significantly reduced^7^. Although both PD and RLS patients had abnormal iron metabolism in brain, the above reports implied that they presented different patterns indicated by the increase of iron in PD and decrease of iron in RLS. So, in PD-RLS patients, how are the levels of iron and related proteins changed in brain? Currently, few studies have detected the levels of iron and related proteins in CSF and serum, and no investigation has focused on the correlation between RLS and iron metabolism in peripheral and central nervous systems in PD patients.

The neurochemical mechanism of RLS is not well elucidated till now. It was found that DA system was damaged indicated by the reduced DA level and the downregulation of D2 receptor in putamen of RLS patients^8^. Additionally, the finding that dopaminergic drugs, the remedies for PD therapy, remarkably alleviated RLS reflected the relationship between dysfunction of DA system and RLS, accordingly, PD and RLS might share similar neurochemical mechanism. Further investigation indicated that the frequency of RLS in PD patients was significantly higher than that in non-PD, indicating a closer association between dysfunction of DA system in SN and RLS. However, part of studies suggested that PD-RLS was relevant to long term of stimulation by dopaminergic drugs^9^. Hence, alteration of DA system in PD-RLS is not determined yet. There was no report about the correlation of PD-RLS with other neurotransmitter systems, such as acetylcholine (Ach), epinephrine (NE) and 5-hydroxytryptamine (5-HT).

In this study, we investigated the clinical characteristics of RLS, the relationship between RLS and motor symptoms or other non-motor symptoms, and explored the potential mechanism involving abnormal iron metabolism and altered neurotransmitters.

## Methods

### Ethics Statement

This study has been approved by Beijing Tiantan Hospital review board. Written informed consent was obtained from all participating subjects. This study was performed according to the guidelines of Capital Medical University, which abides by the Helsinki Declaration on ethical principles for medical research involving human subjects.

### Subjects

Patients were diagnosed with PD according to United Kingdom Parkinson Disease Society Brain Bank criteria^10^. PD patients with systemic diseases, including hypertension, anemia, hepatosis, heart failure, pulmonary disorders, chronic liver/renal failure, severe hypothyroidism and diabetes, and severer dysarthria or mental illness that affect emotional expression were excluded.

We consecutively recruited 218 patients with PD from the Department of Geriatrics and Neurology, Beijing Tiantan Hospital, Capital Medical University from 2012 to 2016. Demographic information, including sex, age, disease duration and education level were recorded.

PD patients were evaluated by diagnostic criteria of the International Restless Legs Syndrome Study Group (IRLSSG), and divided into PD with RLS (PD-RLS) group and PD with no RLS (PD-NRLS) group, respectively. RLS Rating Scale (RLS-RS) was used to evaluate the performance and severity of RLS in PD patients.

### Assessments of clinical symptoms

Severity of PD was evaluated by Hohen and Yahr (H-Y) stage. Motor symptoms of PD were evaluated by the Unified Parkinson Disease Rating Scale (UPDRS) III. Levodopa equivalent does (LED) were calculated for PD patients.

Non-motor symptoms were firstly screened by Non-motor Symptoms Quest followed by series of rating scales, including Hamilton Depression (HAMD) Scale for depression, Hamilton Anxiety (HAMA) Scale for anxiety, Montreal Cognitive Assessment (MoCA) Scale for cognitive impairment, Modified Indifference Rating Scale (MAES) for apathy, Fatigue Severity Scale (FSS) for fatigue, Pittsburgh Sleep Quality Index (PSQI) for sleep disorders, and the Scale For Outcomes in PD For Autonomic Symptoms (SCOPA-AUT) for autonomic dysfunction.

### Collections of CSF and serum

Anti-parkinsonian drugs were withheld for 12–14 hours prior to sampling the CSF. Total 3 ml CSF were obtained by lumbar puncture and 2 ml venous whole blood was collected between 7 a.m. and 10 a.m. under fasting condition, and then placed in a polypropylene tube. Approximately 0.5 ml volume of CSF and serum were aliquotted into separate Nunc cryotubes and kept frozen at −80 °C until ready for assay. Each aliquot dedicated for each measure to avoid freeze-thawing and protein degradation.

### Detections of the levels of iron and related proteins in CSF and serum

The levels of iron and related proteins, including ferritin, transferrin and lactoferrin in CSF and serum from PD patients were detected by Enzyme Linked Immunosorbent Assay. Ab83366 kit for iron, Ab108837 kit for ferritin and Ab108911 kit for transferrin were from Abcam Company (Cambridge, United Kindom). E01L0224 kit for lactoferrin was from Shanghai Lanji Biological Limited Company (Shanghai, China).

### Detections of the levels of neurotransmitters in CSF and serum

The levels of neurotransmitters, including DA, 5-HT, Ach and NE in CSF and serum from PD patients, were measured by high-performance liquid chromatography. Phenomenex 150 × 2 mm, 150 × 3 mm chromatographic column, and liquid chromatography tandem mass spectrometry 6410 instrument were from Agilent (USA), and standard samples were from Sigma (St. Louis, MO, USA).

### Data analyses

Statistical analyses were performed with SPSS Statistics (version 20.0, SPSS Inc., Chicago, IL, USA). P value was significant when it was <0.05.

Demographics variables, motor symptoms, non-motor symptoms and the levels of iron and related proteins and neurotransmitters in CSF and serum were compared between PD-RLS and PD-NRLS groups.

Continuous variables, if they were normally distributed, were presented as mean ± SD deviations and were compared between the 2 groups by using a 2-tailed t test. Bonferroni correction was performed in further comparisons between two groups. Continuous variables, if they were not normally distributed, were presented as median (quartile) and were compared between the 2 groups by using nonparametric test. Discrete variable comparisons were performed by using X^2^ test.

Pearson correlation analyses were performed between RLS-RS score and the level of iron and each related protein in CSF and serum, between RLS-RS score and the level of each neurotransmitter in CSF, between the level of iron and each related protein and neurotransmitter in CSF and serum in PD group.

## Results

### Frequency and assessment of RLS in PD patients

In 218 PD patients, 88 cases (40.37%) had RLS, among which, 53 were male and 35 were female.

We assessed the symptoms of RLS by using RLS-RS and found that the average score of RLS-RS in PD-RLS group was 17.93 ± 0.95 points.

### Demographic information of PD-RLS and PD-NRLS groups

The demographic variables were compared between PD-RLS and PD-NRLS groups, and the data showed a significantly longer disease duration in PD-RLS group than that in PD-NRLS group (Table 1).

**Table 1:** Demographic variables of PD-RLS and PD-NRLS groups.

	PD-RLS group (88 cases)	PD-NRLS group (130 cases)	P value
Age (years, mean ± SD)	61.82 ± 9.23	61.24 ± 8.73	0.65
Male/Total [cases/total (%)]	53/88 (59.49%)	69/130 (53.08%)	0.83
Disease duration [years, median (quartile)]	3.00 (2.00, 5.00)	2.00 (1.00, 4.00)	0.03*
Education level [cases/total (%)]			
Primary school and below	36/88 (40.90%)	34/130 (26.15%)	0.37
Middle and high school	37/88 (42.05%)	63/130 (48.46%)	
Bachelor’s degree and above	15/88 (17.05%)	33/130 (25.38%)	

Two-tailed t test was used for the comparison of age and disease duration between PD-RLS and PD-NRLS groups; X^2^ test was used for the comparison for sex and education level between PD-RLS and PD-NRLS groups. *P < 0.05.

### Comparison of clinical symptoms between PD-RLS and PD-NRLS groups

In 88 PD-RLS patients, 5 cases (5.68%) manifested with RLS before the onset of motor symptoms. PD-RLS group was in more advanced disease stage, and had higher score of UPDRS III than PD-NRLS group (Table 2). Comparison of levodopa equivalent daily dose revealed that PD-RLS group used higher dose of dopaminergic drugs than PD-NRLS group (Table 2).

**Table 2:** Motor symptoms and non-motor symptoms of PD-RLS and PD-NRLS groups.

	PD-RLS group (88 cases)	PD-NRLS group (130 cases)	P value
Motor symptoms			
H-Y stage [stage, median (quartile)]	2.00 (1.50, 2.50)	1.50 (1.00, 2.50)	0.00**
UPDRS III [points, median (quartile)]	26.75 (19.00, 41.75)	20.00 (11.50, 29.00)	0.00**
Levodopa equivalent daily dose [mg, median (quartile)]	1.88 (0.00, 3.75)	0.00 (0.00, 2.50)	0.01*
Non-motor symptoms			
HAMD [points, median (quartile)]	15.00 (9.00, 20.00)	8.00 (3.00, 14.75)	0.00**
HAMA [points, median (quartile)]	11.00 (6.00, 19.00)	6.00 (2.00, 11.00)	0.00**
PSQI [points, median (quartile)]	7.00 (5.00, 10.00)	5.00 (3.00, 9.00)	0.00**
MAES (points, mean ± SD)	18.62 ± 8.32	14.70 ± 9.00	0.01*
FSS (points, mean ± SD)	41.27 ± 20.03	20.78 ± 35.45	0.04*
SCOPA-AUTO score [points, median (quartile)]	11.00 (7.00, 18.00)	9.00 (5.00, 28.25)	0.06
MoCA [points, median (quartile)]	20.00 (15.00, 24.75)	21.50 (16.00, 25.00)	0.23

Two-tailed t test was used for the comparison of scores of MAES and FSS between PD-RLS and PD-NRLS groups; Nonparametric test was used for the comparison of motor symptoms and for the comparison of scores of HAMD, HAMA, PSQI, SCOPA-AUTO score and MoCA. H-Y stage = Hohen and Yahr stage; UPDRS = Unified Parkinson Disease Rating Scale; HAMD = Hamilton Depression Scale; HAMA = Hamilton Anxiety Scale; PSQI = Pittsburgh Sleep Quality Index; MAES = Modified Indifference Rating Scale; FSS = Fatigue Severity Scale; MoCA = Montreal Cognitive Assessment; SCOPA-AUTO = Scale for Outcomes in Parkinson Disease for Autonomic Symptoms. *P < 0.05, **P < 0.01.

Comparison of non-motor symptoms by related rating scales showed that the scores of HAMD, HAMA, PSQI, FSS and MAES in PD-RLS group were significantly higher than those in PD-NRLS group. However, there was no significant difference in the scores of SCOPA-AUTO and MoCA scales between PD-RLS and PD-NRLS groups (Table 2).

### Relationship between PD-RLS and abnormal iron metabolism

**Figure 1:**
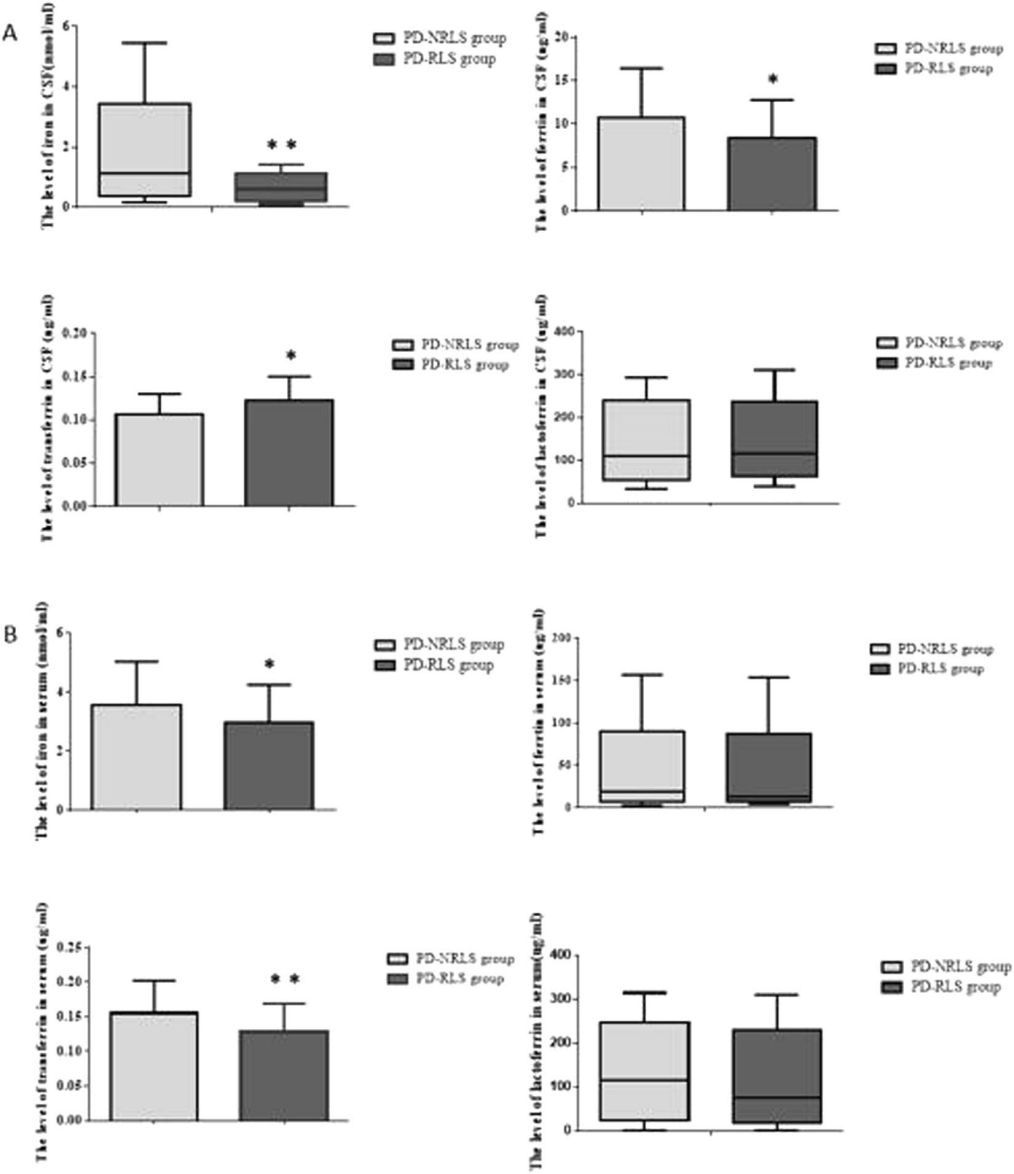
The levels of iron and related proteins in CSF (Fig. 1A) and serum (Fig. 1B) in PD-RLS and PD-NRLS groups. Two-tailed t test was used for the comparison of the levels of ferrtin and transferrin in CSF as well as iron and transferrin in serum between PD-RLS and PD-NRLS groups; Nonparametric test was used for the comparison of levels of iron and lactoferrin in CSF as well as ferritin and lactoferrin in serum between PD-RLS and PD-NRLS groups. *P < 0.05, **P < 0.01.

In CSF, the levels of iron and related proteins, including transferrin, ferritin and lactoferrin were compared between PD-RLS and PD-NRLS groups (Fig. 1A). The data showed that the levels of iron and ferritin were significantly decreased and transferrin level was significantly increased in PD-RLS group compared with PD-NRLS group. Further analyses demonstrated that RLS-RS score was negatively correlated with the levels of iron and ferritin (r = −0.262, P  < 0.05; r = −0.349, P  < 0.01; respectively) and positively correlated with transferrin level (r = 0.250, P  < 0.05) in PD-RLS group.

In serum, the levels of iron and related proteins were compared between PD-RLS and PD-NRLS groups (Fig. 1B). It was observed that the levels of iron and transferrin in PD-RLS group were dramatically decreased when compared with PD-NRLS group. There was no significant difference in ferritin level between the two groups. Further analysis indicated that RLS-RS score was negatively correlated with the levels of iron and transferrin (r = −0.302, P  < 0.05; r = −0.266, P  < 0.01; respectively).

### Relationship between PD-RLS and the levels of neurotransmitters in CSF

**Figure 2:**
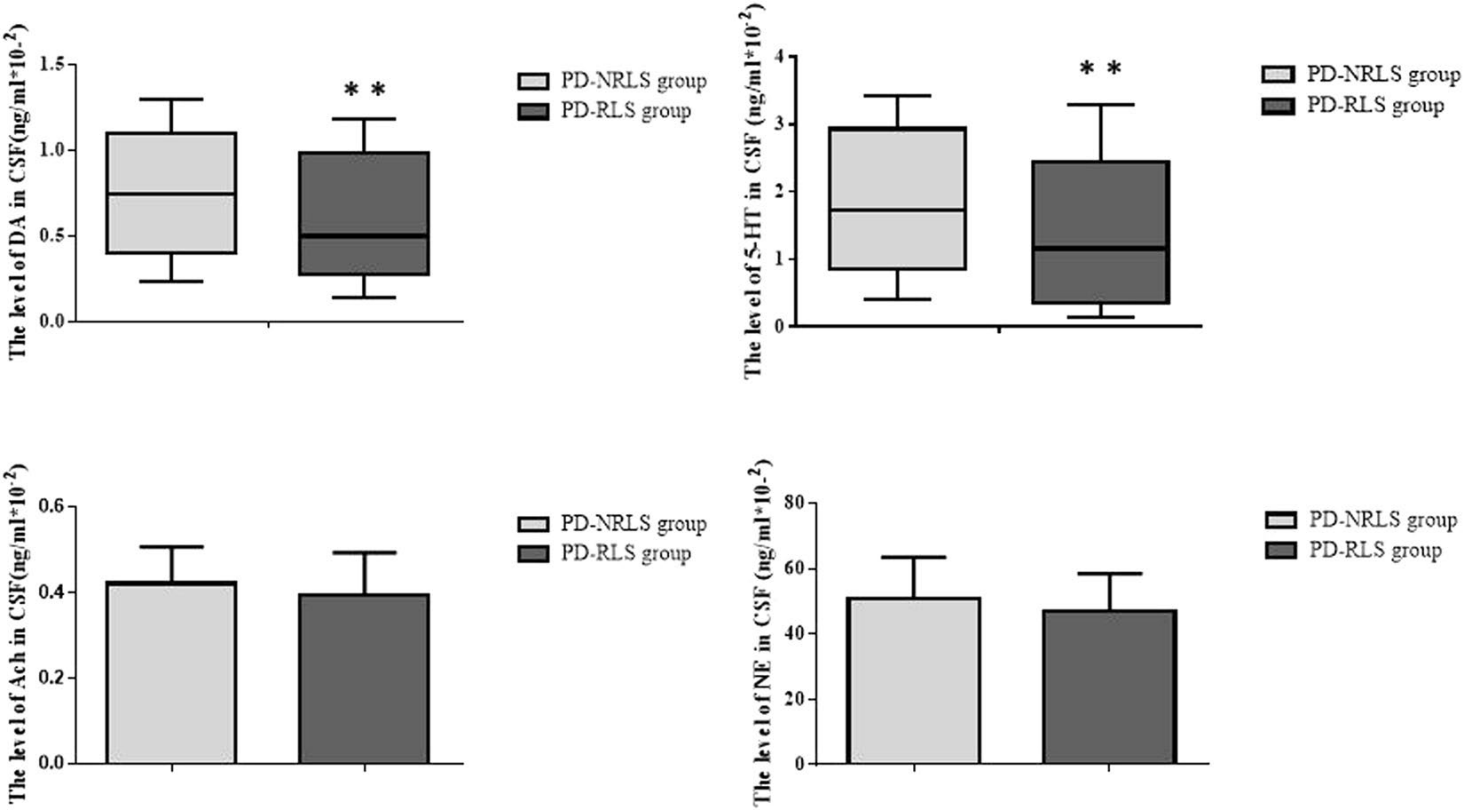
The levels of neurotransmitters in CSF of PD-RLS and PD-NRLS groups. Two-tailed t test was used for the comparison of the levels of acetylcholine (Ach) and adrenaline (NE) in CSF between PD-RLS and PD-NRLS groups; Nonparametric test was used for the comparison of the levels of dopamine (DA) and 5-hydroxytryptaphanein (5-HT) in CSF between PD-RLS and PD-NRLS groups. **P < 0.01.

Compared with PD-NRLS group, the levels of DA and 5-HT in CSF in PD-RLS group were drastically decreased (Fig. 2). The levels of Ach and NE in CSF were not different between the two groups (P > 0.05).

Further analysis suggested that RLS-RS score was negatively correlated with the levels of DA and 5-HT in CSF (r = −0.448, P  < 0.01; r = −0.333, P  < 0.01; respectively).

### Relationship between the levels of iron and related proteins and neurotransmitters in CSF

In PD-RLS group, we analyzed the relationship between the levels of iron and related proteins and neurotransmitters and observed that ferritin level was positively correlated with DA level in CSF (r = 0.225, P  < 0.05) and transferrin level was negatively correlated with the levels of DA and 5-HT in CSF (r = −0.395, P  < 0.05; r = −0.230, P  < 0.05; respectively).

## Discussion

The research of PD-RLS was relatively sufficient compared with other non-motor symptoms of PD. RLS has been paid much attention since the International Restless Legs Syndrome Study Group (IRLSSG) published the RLS diagnostic criteria in 2003^11^. In this study, we evaluated the frequency of RLS, assessed the symptoms of RLS, and investigated demographic features, motor symptoms and non-motor symptoms of PD-RLS patients.

In PD patients, we found that the frequency of RLS was 40.37%, suggesting that RLS was one of the common non-motor symptoms of PD. It was reported that the incidence of RLS in PD patients was ranged from 0.98% to 16% in east Asian population^12^. The higher frequency of PD-RLS in this study than that in previous studies^13^ might be due to the differences in the age and disease duration of PD population.

RLS-RS was used to evaluate the performance and severity of RLS in PD patients. We observed that the symptoms of RLS were mild to moderate according to RLS-RS scoring criteria, which might be because that most of the recruited PD patients were in their early disease stage with an average H-Y stage of 1.96, or received dopaminergic drugs, leading to the reduction of RLS.

Comparisons of demographic variables displayed no significant difference in age, sex, disease duration and education level (Table 1) between PD-RLS and PD-NRLS groups, demonstrating that above demographic information was not related to RLS. In this study, PD-RLS group had longer disease duration than PD-NRLS group, which might be because that RLS worsened with the depletion of DA with time extending.

RLS could occur in the second stage of PD pathological stage by Braak^14^, suggesting that RLS was one of premotor symptoms of PD. We found that 5 out of 88 PD-RLS patients were with RLS with a frequency of 5.68% before the onset of motor symptoms, which was lower than previously reported results. We speculated that PD patients in this study did not pay enough attention to RLS before the onset of motor symptoms.

Our results showed that H-Y stage in PD-RLS group was more advanced than that in PD-NRLS group, implying that the disease status, indicated mainly by the motor function, of PD -RLS group was more severe than that of PD-NRLS group. Additionally, UPDRS III score in PD-RLS group was significantly higher than that in PD-NRLS group, suggesting that PD-RLS group had more serious motor symptoms than PD-NRLS group^15^. Studies found that the severity of motor symptoms of PD patients was related with RLS, implying that motor symptoms and RLS might be correlated with the dysfunction of DA system. When PD stepped into an advanced stage, the synthesis of DA was furtherly reduced, which accordingly led to the aggravation of RLS^16^.

Levodopa equivalent daily dose in PD-NRLS group was dramatically higher than that in PD-NRLS group, reflecting that PD-RLS group might has insufficient DA level due to severer disease status and consequently used high dose of dopaminergic drugs to alleviate clinical symptoms.

Depression and anxiety are the two common mood disorders in PD patients. In 126 patients with PD, Krishnan found that the incidence of depression in PD-RLS group was higher than that in PD-NRLS group^17^. Additionally, a previous study showed that the anxiety of PD-RLS group was severer than that of PD-NRLS group^18^. In this study, compared to PD-NRLS group, PD-RLS group presented more serious depression and anxiety indicated by the dramatic increased sores of HAMD and HAMA, respectively. It was found that depression and anxiety were associated with the decreased 5-HT level in brain^19^. However, the potential mechanism of depression and anxiety in PD-RLS patients remains unclear. We furtherly observed that 5-HT level in CSF in PD-RLS group was significantly lowered than that in PD-NRLS group, implying that dysfunction of 5-HT system might be a potential neurochemical mechanism underlying depression and anxiety in PD-RLS patients.

Multiple sleep disorders, such as dysfunction of sleep rhythms during night and an excessive sleepiness in the daytime, are seen in PD patients. The incidence of sleep disorders in PD patients was almost up to 70%^19^. This investigation showed that, compared with PD-NRLS group, PD-RLS group had seriously compromised sleep quality indicated by the evidently increased PSQI score. We then explored the potential mechanism of bad sleep quality and found that DA level in CSF in PD-RLS group was much lower than that in PD-NRLS group, implying that DA system was fairly compromised in PD patients with RLS. Dysfunction of mesocorticolimbic DA system caused sleep disorders with typical manifestations of lack of normal rhythms during night time and an excessive sleepiness during daytime^20^, therefore, severer sleep disorders and poorer sleep quality might be related to the seriously decreased DA level in brain.

Fatigue is a frequently ignored non-motor systems of PD, which was thought to be one of the most critical factors causing disabling and reduction of life quality in PD patients. We demonstrated that, compared with PD-NRLS group, PD-RLS group suffered from evident fatigue indicated by the dramatically increased FSS score. A previous study suggested that, similar to depression and anxiety, fatigue might also be related to the dysfunction of 5-HT system^21^. In this study, 5-HT levels in CSF in PD-RLS group was significantly reduced compare with that in PD-NRLS group, implying the impairment of 5-HT system might be closely related to the fatigue in PD-RLS subjects^22^.

Apathy is one of the common psychiatric symptoms of PD. The relationship between RLS and apathy in PD patients has not been reported yet. In this study, PD-RLS group presented more serious apathy reflected by the dramatically increased score of MAES scale compared with PD-NRLS group. Apathy was found to be related to the impaired function of anterior frontal, orbital and striatum ventral loop^23^, suggesting that depletion of DA in brain might be pivotal for apathy^24^. Meanwhile, it was observed that RLS was also associated with impaired DA system. Hence, we speculated that RLS and apathy might have overlapping neurochemical mechanism in PD patients.

When we found the correlation between above non-motor symptoms and RLS, we failed to observe the significant association between other non-motor symptoms, such as autonomic dysfunction and cognitive impairment, and RLS in PD patients.

α-synuclein oligomer is the major component of Lewy bodies, the pathological hallmark of PD, which was found in the structures relevant to autonomic dysfunction, including peripheral autonomic nervous system, autonomic nervous system of spinal cord and medulla, before the onset of motor symptoms in PD patients^25^. A recent study reported the existence of autonomic symptoms, such as nocturnal/supine hypertension and blood pressure fluctuations, in PD patients, suggesting the correlations between RLS and autonomic impairment^26^. Here, we displayed a tendency of worsened autonomic symptoms in PD-RLS group compared with PD-NRLS group. It is needed to enhance the sample size to explore the relationship between PD-RLS and autonomic impairment.

Cognitive impairment is a non-motor symptom that markedly reduces the quality of life for PD patients. Cognitive impairment, particularly, the severer cognitive impairment occurs in the later stage of PD, which is mainly related to the dysfunction of cholinergic system. In this exploration, PD-RLS and PD-NRLS groups showed no significant difference in MoCA score and Ach level in CSF, both of which were not correlated with PD-RLS through further analyses. These data indicated that cognitive impairment was not related to PD-RLS both in clinical and neurochemical aspects, which might be because that most of PD patients recruited were in the early and middle stages of PD, and easily suffered from RLS and dysfunction of DA system; whereas, cognitive impairment and depletion of Ach occurred in the late stage of PD.

Both PD and RLS are associated with the abnormalities of iron metabolism. In PD patients, it was found that iron level was significantly increased in 1924 by Lehermitte. Subsequently, a large number of histopathological studies and autopsy results showed a large amount of iron deposition in SN of PD patients, in which the total iron content was increased from 25% to 100% and the iron ion content was increased by 225%^27, 28^. Our previous *in vivo* study found that iron caused selective and progressive dopaminergic neurodegeneration, and microglial activation potentiated the neurotoxicity^29^. In RLS patients, it was observed that insufficient iron in brain might be the potential mechanism underlying RLS^30^. However, the pathogenesis of PD-RLS is not well understood.

We detected the levels of iron and related proteins in PD patients. We firstly found that iron levels in CSF in PD-RLS group were drastically decreased compare with that in PD-NRLS group, implying a potential relationship between brain iron dysfunction and PD-RLS. Nigrostriatal pathway is one of the main regions of iron deposition in brain^31^, therefore, iron might be insufficient in RLS-related regions when it was excessively deposited in nigrostriatal pathway, which might be relevant to RLS in a part of PD patients. Further analyses showed that iron level in CSF in PD-RLS group was negatively correlated with RLS-RS score, which demonstrated that the lower the iron level in brain, the severe the degree of PD-RLS. Thus, iron level in brain might reflect the severity and progression of RLS for PD patients. In serum, we observed that the iron level in PD-RLS group was also significantly lowered compared with PD-NRLS group, and iron level was also negatively correlated with RLS-RS score, indicating that the iron level in peripheral system was also significantly reduced, similar to the alteration in brain.

Transferrin is a receptor-mediated transporter of iron from peripheral system to brain across the blood-brain barrier^32^, which exerts critical action on iron metabolism, particularly, iron transport, and participates in maintaining iron homeostasis. In normal condition, iron (mainly Fe^3+^) absorbed from gastrointestinal tract into blood enters brain across BBB in a manner of transferrin receptor-mediated transport. In this investigation, transferrin level in CSF in PD-RLS group was significantly higher than that in PD-NRLS group, and transferrin level in CSF had a significantly positive correlation with RLS-RS score, based on which, we speculated that transferrin might take substantial amount of iron from peripheral system into brain. (However), iron level in CSF in PD-RLS group was remarkably decreased, so, iron might not be transported to the regions related to PD-RLS, which might decrease iron level in RLS-related regions, and thus was associated with RLS in PD patients. In serum, transferrin level in PD-RLS group was obviously reduced compared with PD-NRLS group, meanwhile, transferrin level in CSF in PD-RLS group was drastically elevated compared with PD-NRLS group, which furtherly supported that in PD-RLS group, more transferrin in peripheral blood carried iron into brain. Subsequent analyses exhibited a significantly negative correlation between transferrin level in serum and RLS-RS score, revealing that the lower the transferrin level in serum, the severe the degree of PD-RLS. These data suggested a disturbed hemostasis of iron transport in peripheral system, which pattern was contrary to that in the brains of PD-RLS.

Ferritin is mainly in charge of binding and storage of iron, and plays an important role on maintaining homeostasis of iron metabolism. Ferritin can dynamically regulate iron content in brain, avoiding neurotoxicity produced when iron is excessively deposited and serving as a source when iron is prominently reduced. In this study, ferritin level in CSF in PD-RLS group was obviously decreased compared with PD-NRLS group. Further analysis indicated a significant negative correlation between ferritin level in CSF and RLS-RS score. These results supported the hypothesis that reduction of ferritin level in part of regions was pivotal for PD-RLS, which might be explained that insufficient ferritin failed to properly regulate the process of iron metabolism and induced iron deficiency^7, 33^ in the regions relevant to RLS in PD brains. The detailed mechanism needs to be investigated profoundly in the future.

Both PD and RLS are associated with the dysfunction of DA system in brain. In PD patients, DA system in nigrostriatual pathway is mainly involved, which is featured by the progressive degeneration and death of DA neurons in SN and subsequently dramatic depletion of DA in striatum. In RLS patients, multiple DA systems, including SN, striatum, diencephalon, hypothalamus and spinal cord^34^, were demonstrated to be associated with the dysfunction of DA system, in which, DA mainly plays an inhibitory effect on the excitability of spinal cord^35^, thus decrease of DA synthesis might increase the excitability of spinal cord, leading to the occurrence of RLS. However, few investigations focus on the location and neurochemistry of PD-RLS.

As for the location relevant to PD-RLS, studies demonstrated that basal ganglia could be involved^36^. The significant DA reduction in brain is seen both in PD and RLS patients. However, there is no consistent conclusion about DA alteration in the brains of PD-RLS patients. A previous study has found that levodopa could effectively improve the symptoms of RLS, which demonstrated indirectly that DA deficiency might be related to RLS^16^. Another investigation by using SPECT scan observed that PD-RLS group had an evidently high binding rate of tracer with DA transporter within 4 years of disease onset^37^, which also indirectly reflected a lower brain DA level in PD-RLS subjects. However, a study showed that longtime use of dopaminergic drugs could be one of the reasons of PD-RLS^9^. In this study, we directly measured DA level in CSF and found an evident DA reduction in PD-RLS group compared with that in PD-NRLS group. Furthermore, DA level in CSF was negatively correlated with PD-RLS score, indicating that PD-RLS was highly relevant to DA deficiency in brain. These data provide the direct evidence of potential neurochemical mechanism of PD-RLS.

Based on the fact that transferrin level in CSF was significantly increased and iron level in CSF was significantly decreased in PD-RLS group compared with PD-NRLS group, we speculated that iron transported from the peripheral system to brain might manly deposit in SN, leading to progressive degeneration and death of DA neurons and subsequent DA depletion in striatum. Meanwhile, we know that iron is a cofactor of tyrosine hydroxylase, the rate-limiting enzyme of DA synthesis. If iron is insufficient in other brain regions, such as those related to RLS, DA synthesis will be reduced. Accordingly, we speculated that, in part of PD patients, excessive iron-caused damage to SN and subsequent depletion of DA in the striatum, and insufficient iron-reduced DA synthesis in RLS-related brain regions would significantly decrease DA level, which was related to PD-RLS.

Further analyses indicated that the higher the transferrin level in CSF and the lower the ferritin level in CSF, the lower the DA level in CSF; meanwhile, the lower the DA level in CSF, the severer the symptoms of RLS. These results implied that abnormal iron metabolism, including the transport, binding and storage of iron, might decrease DA level in brain through above multiple potential mechanisms. Additionally, RLS was progressively exacerbated with DA level was sustainably depleted. Consequently, DA deficiency might be a pivotal neurochemical mechanism of PD-RLS.

Currently, there is less investigation on the relationship between RLS and 5-HT. Population and case control studies, and case reports showed that 5-HT reuptake inhibitors might aggravate or alleviate the symptoms of RLS^38^. Hence, relationship between RLS and 5-HT is not clearly elucidated. Based on the facts that 5-HT could promote or inhibit DA activity through several subtypes of its receptors^39^, and the DA deficiency was related to PD-RLS, accordingly, we hypothesized that 5-HT might be related to PD-RLS through interacting with DA system. Indeed, we found that 5-HT level in CSF in PD-RLS group was robustly declined than that in PD-NRLS group, and further correlation analysis exhibited a significantly negative correlation between RLS-RS score and 5-HT level in CSF. These findings suggested dysregulation of 5-HT system might provoke or exacerbate the pathogenesis of PD-RLS.

In patients with PD-RLS, more brain regions were extensively involved and non-motor symptoms, such as depression, anxiety and fatigue were more severer, which were associated with the decrease of 5-HT level^39^. Based on the finding that PD-RLS was related to iron metabolism, we then analyzed the relationship among the levels of iron and related proteins in CSF, the level of 5-HT in CSF and the score of RLS in PD-RLS patients. The results demonstrated that the higher transferrin level was correlated with the lower 5-HT level in CSF, and symptoms of RLS were aggravated as 5-HT level in CSF was decreased in PD-RLS patients. Thus, abnormalities of iron metabolism, particularly, dysfunction of iron transport, was more likely related to the reduction of 5-HT, which might serve as another neurochemical mechanism of PD-RLS^40^.

We also detected the levels of Ach and NE in CSF and serum, and no significant difference was revealed between PD-RLS and PD-NRLS groups. Hence, Ach and NE were not relevant to PD-RLS.

We need to mention that the level of iron, each related protein and neurotransmitter in CSF and serum might be related to age, duration of the disease, and the medication used. Thus, multiple linear regression models were established separately, and the relationships between RLS score and the level of iron, each related protein in CSF and serum or each neurotransmitter in CSF were adjusted with age, disease duration and the medication used. The results showed that RLS score was still significantly and negatively correlated with ferritin level and positively correlated with transferrin level in CSF, dramatically and negatively correlated with the levels of iron and transferrin in serum, prominently and negatively correlated with the levels of DA and 5-HT in CSF. (Supplementary Table 1). After the adjustment, RLS score was negatively correlated with iron level in CSF, and the P value was very close to be significant (Supplementary Table 1), which might be accounted for the insufficient sample size due to the difficulty of collecting CSF from elderly PD patients. Currently, we are still recruiting PD patients and increasing CSF samples to testify the significant correlation between RLS and iron level in CSF.

In summary, the frequency of PD-RLS was high. PD-RLS patients were in more advanced stage of disease, had severer motor symptoms and part of non-motor symptoms, including depression, anxiety, sleep disorders, fatigue and apathy. Dysfunctions of iron transport, binding and storage in the peripheral and central nervous systems mediated iron deficiency in the brain regions relevant to RLS, which was related to PD-RLS through decreasing the levels of DA and 5-HT in brain (results) from this investigation provide evidence for elucidating clinical characteristics and exploring therapeutic strategy for PD-RLS.

## Electronic supplementary material

Supplementary Information
