# Supplementary material for: Restless legs syndrome in Parkinson disease: Clinical characteristics, abnormal iron metabolism and altered neurotransmitters
Source: Sci Rep. 2017 Sep 5;7:10547. doi: 10.1038/s41598-017-10593-7 (PMC5585207; doi:10.1038/s41598-017-10593-7)
Supplement: Supplementary file 1 — Supplementary Information [file 41598_2017_10593_MOESM1_ESM.doc]

**Restless legs syndrome in Parkinson disease:**

**Clinical characteristics, abnormal iron metabolism and altered neurotransmitters**

Ying-Shan PIAO^1^, Teng-Hong LIAN^2^, Yang HU^1^, Li-Jun ZUO^2^, Peng GUO^1^, Shu-Yang YU^1^, Li LIU^1^, Zhao JIN^2^, Hui ZHAO^1^, Li-Xia LI^1^, Qiu-JinYU^2^, Rui-Dan WANG^2^, Sheng-Di CHEN^7^, Piu CHAN^8,5,6^, Xiao-Min WANG^9,4,5,6^, Wei ZHANG^1, 2, 3, 4, 5,6^

1 Department of Geriatrics, Beijing Tiantan Hospital, Capital Medical University, Beijing, 100050, China

2 Department of Neurology, Beijing Tiantan Hospital, Capital Medical University, Beijing, 100050, China

3 China National Clinical Research Center for Neurological Diseases, Beijing, 100050, China

4 Key Laboratory for Neurodegenerative Disorders of the Ministry of Education, Capital Medical University, Beijing, 100069, China

5 Center of Parkinson Disease, Beijing Institute for Brain Disorders, Beijing, 100069, China

6 Beijing Key Laboratory on Parkinson Disease, Beijing, 100053, China

7 Department of Neurology, Ruijin Hospital Affiliated to Shanghai Jiaotong University School of Medicine, Shanghai, 200025, China

8 Department of Neurobiology, Beijing Xuanwu Hospital, Capital Medical University, Beijing, 100053, China

9 Department of Physiology, Capital Medical University, Beijing, 100069, China

**Corresponding author:** Wei ZHANG, MD, PhD, Department of Geriatrics, Department of Neurology, Beijing Tiantan Hospital, Capital Medical University, Beijing,100050, China; China National Clinical Research Center for Neurological Diseases, Beijing, 100050, China; Key Laboratory for Neurodegenerative Disorders of the Ministry of Education, Capital Medical University, Beijing, 100069, China; Center of Parkinson Disease, Beijing Institute for Brain Disorders, Beijing, 100069,China; Beijing Key Laboratory on Parkinson disease，Beijing, 100053, China Telephone: +8613911996107; Fax: 86-10-67098429; E-mail: [ttyyzw@163.com](mailto:ttyyzw@163.com.cn)

This work was performed at Beijing Tiantan Hospital, Capital Medical University, Beijing, China

|  | **β** | **p value** |
| --- | --- | --- |
| Iron level in CSF | -0.400 | 0.08 |
| Ferritin level in CSF | -1.330 | 0.034* |
| Transferrin level in CSF | 174.79 | 0.039* |
| Iron level in serum | -2.340 | 0.038* |
| Transferrin level in serum | -122.77 | 0.002** |
| Dopamine level in CSF | -2214.92 | 0.006** |
| 5-hydroxytryptaphane level in CSF | -501.134 | 0.025* |

Supplementary Table 1 Linear regression analyses between RLS score and the level of iron, each related protein in CSF and serum or each neurotransmitter in CSF in PD group. *P＜0.05，**p＜0.01.
